# Heavy Metal Hazards of Pediatric Syrup Administration in Nigeria: A Look at Chromium, Nickel and Manganese

**DOI:** 10.3390/ijerph6071972

**Published:** 2009-07-09

**Authors:** John Kanayochukwu Nduka, Orish Ebere Orisakwe

**Affiliations:** 1 Environmental Chemistry and Toxicology Research Unit, Pure and Industrial Chemistry Department, Nnamdi Azikiwe University, P.M.B. 5025, Awka Anambra State, Nigeria; E-Mail: johnnduka2000@yahoo.co.uk; 2 Toxicology Unit, Department of Pharmacology, Nnamdi Azikiwe University, College of Health Sciences Nnewi Campus, Nnewi, Anambra State, Nigeria

**Keywords:** chromium, nickel, chromium, pediatric syrups, pollution, public health, Nigeria

## Abstract

Fifty different pediatric syrups were randomly sampled from patent medicine stores and pharmaceutical shops within Awka (Anambra State, Nigeria) between November 2007 and May 2008. Syrups were ashed before digestion using conc. aqua regia, HCl: HNO_3_ (3:1). Chromium, nickel and manganese were assayed with AAS 205A. The highest levels of nickel were seen in Magcid suspension (4.13 mg/L) and Gaviron (0.79 mg/L) whereas lowest levels were found in Asco–J vitamin and Jawaron Syrup (0.01 mg/L). About 44.1, 73.6 and 20.6% of the sampled syrups made in Nigeria had non detectable levels of nickel, chromium and manganese respectively. Chromium levels ranged from 0.01 mg/L in Magcid suspension to 0.58 mg/L in emvite. Ferobin and Jawaron Syrup plus had 28.23 and 4.37 mg/L manganese, respectively. With the exception of Cephalexin Syrup, all the imported syrups had non detectable levels of chromium. The level of chromium in Cephalexin Syrup was 0.01 mg/L. About 68.8 and 43.7% of these imported syrups had non-detectable levels of nickel and manganese respectively. Nickel levels ranged from 0.01–0.09 mg/L in the imported syrups. Haemoglobin Syrup showed highest level of manganese of 0.36 mg/L whereas the lowest concentration was 0.02 mg/L in Cadiphen. Taken together the Nigerian made syrup samples had higher concentrations of the studied heavy metals. It is feared that ingestion of these syrups may constitute a significant source of heavy metal exposure to the children and should therefore be considered a public health problem. The public health hazards from ingestion of these syrups should be identified and disclosed by in-depth risk assessment studies.

## 1. Introduction

The health effects of chemical contaminants in consumables are a major health concern today. A very vulnerable and sensitive time in human development is in the womb and during the first five years of infancy. Unfortunately, it is during this time that we take between 70 and 80% of the toxicants accumulated by the body during our lifetimes [1]. In a recent biomonitoring survey of heavy metals levels in children aged 2–6 years in Nigeria by Nriagu and coworkers, many heavy metals ranging from chromium, nickel, manganese, including the traditional offenders like lead and cadmium to radioactive elements were found [2]. Whereas the sources of the lead and cadmium are traceable to common matrices like food, water, air, etc, the sources of the other metals were a mystery.

In a prevalence study of parental medication on children before going to hospital performed by Orisakwe *et al.* 1994 [3], 99% of children aged between 1–6 years were found to have received it before seeing a doctor. About 39% of the studied population had taken at least two drugs before been taken to the hospital. The most frequently administered drugs were paracetamol and chloroquine. Home treatment may be beneficial, but more often these drugs were taken concurrently or consecutively. Drug and non drug poisoning have been reported amongst children in Nigeria. Since there are no Poison Control centers in Nigeria, there are no standard therapeutic modalities in poison management. What exists is largely conservative management which employs all kinds of syrups in a poly-pharmacy manner in symptomatic management of poisoning even in the hospitals [4]. These have led to high patronage of the pediatric syrups either by the parents or the hospitals.

In an attempt to have capture of the possible sources of heavy metals in children in Nigeria, the present study is aimed at investigating the levels of heavy metals like nickel, chromium and manganese in pediatric syrups commonly sold as over-the-counter drugs OTC. Assessment of heavy metal levels in commonly prescribed pediatric drugs in Nigeria where there is paucity of data is worthwhile. It is advocated that any legislation to check heavy metals exposure to humans should be based on genuine scientific evaluation of the available data [5]. Our aim of carrying out this study is to investigate the heavy metal (chromium, nickel and manganese) levels of pediatric syrups sold in Nigeria, which are highly consumed especially by children.

## 2. Materials and Method

Using a market basket protocol fifty pediatric syrups (one per sample) purchased from patent medicine stores and pharmaceutical shops in Awka, the Anambra State capital, Nigeria, were used for the study. The pediatric syrups were divided into two groups of Nigeria made and imported syrups. The samples were ashed and digested in Teflon labware that had been cleaned in a high-efficiency particulate air (HEPA) filtered (class 100), trace-metal-clean laboratory to minimize contamination. This protocol involved sequential cleaning of the labware in a series of baths in solutions (1 week each) and rinses (five per solution) in a three-step order, namely a detergent solution and deionized water rinses, then 6 N HCl (reagent grade) solution and ultra-pure water rinses, finally 7.5N HNO_3_ (trace metal grade) solution and ultra pure water rinses. The labware was then air dried in a polypropylene laminar air flow-exhausting hood. Dry ashing method was used by adding 30 mL of each sample into a conical flask and heated on a hot plate at 200 °C, for 45 mins, then in a furnace at 500 °C until the volume was drastically reduced to near dryness. Digestion was done by addition of 10 mL conc. aqua regia (3:1 HCl: HNO_3_), it was then heated to dryness. Twenty ml de-ionized water was added, stirred and filtered. The filtrate was made up in standard volumetric flask. Nickel, chromium and manganese were assayed with a 205A Atomic Absorption Spectrophotometer with detection limit of 0.001. The background level found in a blank container was 0.001 mg/L. The true intake using the arithmetic mean according to [6] was calculated by multiplying contaminant level i.e., heavy metal level by amount/ volume of syrup. In all the estimated or calculated levels of chromium, nickel and manganese in the syrups, 5 mL was assumed to be the average volume for all the syrups.

## 3. Results and Discussion

Table 1 shows the nickel, chromium and manganese levels of Nigerian made pediatric syrups. The highest levels of nickel were seen in Magcid Suspension (4.13 mg/L) and Gaviron (0.79 mg/L), whereas lowest levels were found in Asco –J vitamin and Jawaron Syrup (0.01 mg/L). About 44.1% of the sampled syrups made in Nigeria had non detectable levels of nickel. Chromium levels ranged from 0.01 mg/L in Magcid Suspension to 0.58 mg/L in Emvite. However 73.6% of the samples made in Nigeria had non detectable levels of chromium. Only 20.6% of the Nigerian made syrups had non detectable levels of manganese. Ferobin and Jawaron Syrup plus had 28.23 and 4.37 mg/L manganese respectively.

The heavy metal (Cr, Ni and Mn) levels in imported syrups is shown on Table 2. With the exception of Cephalexin Syrup, whose level was 0.01 mg/L, all the imported syrups had non detectable levels of chromium. About 68.8% of these imported syrups had non-detectable levels of nickel. Detectable nickel levels in the imported syrups ranged from 0.01–0.09 mg/L. Haemoglobin Syrup showed highest level of manganese of 0.36 mg/L whereas the lowest concentration was 0.02 mg/L in Cadiphen. Only 43.8% of the imported syrups had non-detectable levels of manganese. Taken together the Nigerian made syrup samples had higher concentrations of chromium, nickel and manganese.

The estimated or the calculated intake of chromium, nickel and manganese is contained in Table 3. The calculated amounts of chromium, nickel and manganese in the three most likely administered syrups (Ferobin plus 0.03, ND, and 28.23 of Ni, Cr and Mn respectively), Magcid Suspension (4.13, 0.01 and 1.90 of Ni, Cr and Mn, respectively) and Gaviron (0.79, 0.08 and 1.64 of Ni, Cr and Mn, respectively) with volume 5 mL each were 25.6 mg/mL, Ni, 0.9 mg/mL, Cr, and 158.85 mg/mL, Mn.

This study has investigated the concentration of three potent environmental toxicants known to be potent neurotoxins in commonly used pediatric syrups in Nigeria. We are not aware of any previous investigation of the levels of chromium, nickel and manganese in pharmaceuticals in Nigeria. The highest levels of nickel were seen in Magcid suspension (4.13 mg/L) and Gaviron (0.79 mg/L) whereas lowest levels were found in Asco –J vitamin and Jawaron Syrup (0.01 mg/L). About 44.1, 73.6 and 20.6% of the sampled syrups made in Nigeria had non detectable levels of nickel, chromium and manganese respectively. Chromium levels ranged from 0.01 mg/L in magcid suspension to 0.58 mg/L in emvite. Ferobin and Jawaron Syrup plus had 28.23 and 4.37 mg/L manganese respectively. With the exception of cephalexin syrup all the imported syrups had non detectable levels of chromium. The level of chromium in Cephalexin Syrup was 0.01 mg/L. About 68.8 and 43.7% of these imported syrups had non-detectable levels of nickel and manganese respectively. Nickel levels ranged from 0.01–0.09 mg/L in the imported syrups. Haemoglobin Syrup showed highest level of manganese of 0.36 mg/L whereas the lowest concentration was 0.02 mg/L in Cadiphen. The presence of high levels of manganese is of importance with respect to its possible role in autism, an emerging public health problem which has attracted public discourse only recently.

In Chinese children, exposure to elevated manganese concentrations in drinking water was associated with lower scores on tests of short-term memory, manual dexterity, and visual perceptual speed [7]. Woolf *et al.* reported a child with manganism (blood Mn 3.8 μg/dL) from a private well water source with normal full scale IQ but deficits in verbal, visual and general memory indices [8]. Using the McCarthy General Cognitive Index test at age 5 years, Takser *et al.* [9] reported deficits in memory, attention and psychomotor indices associated with elevated umbilical cord Mn levels. More recently, inverse associations between water Mn levels and IQ among ten year old children has been found. The calculated amount of chromium, nickel and manganese in three most likely administered syrups Ferobin plus, Magcid Suspension and Gaviron each were 25.6 mg/mL, Ni, 0.9 mg/mL, Cr, and 158.85 mg/mL, Mn. If upon a single administration this amount of heavy metals gains entry into the body, the implication is that pediatric syrup could add to the body burden of heavy metals in Nigeria. For a child with compromised health status who has already been exposed through other environmental sources like foods and beverages [10], the public health consequences can be grave.

Why are children most vulnerable to neurotoxins? During fetal life and early childhood, neurons must undertake migration, synaptogenesis, selective cell loss, myelination and a process of selective synaptic pruning before development is complete [11]. Even minor inhibitory or excitatory signals imposed by environmental toxicants at early stages of CNS development can therefore cause alterations to subsequent processes. The nature of CNS development limits the capacity of the developing brain to compensate for cell loss or disruptions in neural networking caused by neurotoxic chemicals and can lead to reductions in cell numbers [12] or alterations in synaptic architecture [13].

In Nigeria, it is common practice for doctors to recommend most of these drugs known to cure specific ailments (such as indigestion, headache, malaria, cough, measles, cold, catarrh, anaemia, stomach upset etc) to pregnant women and lactating mothers and children. A possible route of these metals into these drugs may be during processing such as lead solder, use of contaminated water, poor assaying of raw materials, packaging, poor hygiene and storage facilities. Multivitamins and mineral preparations are widely used for infants and children. All of these preparations contain a variety of excipients (“inert ingredients”). Excipients are generally safe; however, adverse effects have been attributed to them. Complete information about the excipients in various preparations is not readily available. The mandatory listing of all excipients is the only way to assure that physicians and consumers will be fully informed about the hidden ingredients [14].

Heavy metals may just be one of the several contaminants in pediatric syrups either produced or imported into Nigeria, several deaths were reported in Nigeria recently as a result of usage of propylene glycol contaminated with diethylene glycol in production of ‘*my pikin teething*’ paracetamol syrup by a local pharmaceutical company [15]. The presence of metals in seven key herbal mixture is of serious concern, as this drug is in high demand in Nigeria for treating measles.

The Nigerian made syrup samples had higher concentrations of chromium, nickel and manganese. One fact which is evident in this study is that all the pediatric syrups were duly registered by the Food and Drug regulatory agency. It could therefore be inferred that heavy metals are not regulated in medicaments in Nigeria unlike most other countries. Therefore, it is expedient in the interest of public health to introduce mandatory testing for heavy metals for every batch of drug that is produced or imported into the country. Permissible limits for these heavy metals will be as recommended by WHO publication. Conspicuous display on the container or packaging of these medicines should bear the inscriptions like “HEAVY METALS WITHIN PERMISSIBLE LIMITS”.

## 4. Conclusions

It is feared that ingestion of these syrups may constitute a significant route of heavy metal exposure to the children and should therefore be considered a public health problem especially with over dosages arising from self prescription by care givers and parents. The public health hazards from ingestion of these syrups should be identified and disclosed by in-depth risk assessment studies.

## Figures and Tables

**Table 1.** Ni, Cr and Mn Levels (mg/L) of Nigerian made pediatric syrups.

**S/No**	**Pharmaceutical Product**	**Batch No**	**NAFDAC NO**	**MFG Date**	**Expiry Date**	**Metal level (mg/L)**			**Place of Mfg.**
						**Ni**	**Cr**	**Mn**	

1	Asco –J vitamin	0020	04-7233	Aug. 2007	Aug. 2009	0.01	0.37	0.04	Enugu
2	Emvite	4110k	04-0135	Aug. 2007	Aug. 2010	0.07	0.58	0.15	Lagos
3	J - Vite	0018	04-7232	Jul. 2007	Jul. 2009	0.09	0.27	0.06	Enugu
4	Ferobin plus	LA79138	04-4838	Jun. 2007	May 2010	0.03	nd	28.23	Ogun
5	Emzolyn	3050k	04-1454	May 2007	May 2010	nd	nd	0.05	Lagos
6	Multivite	7f434012	04-0331	Jun. 2007	Nov. 2008	nd	nd	0.01	Ogun
7	Kingsize Vit C	HM2279C	04-0879	Jan 2006	Dec. 2008	0.02	0.02	0.02	Ogidi
8	Asco-J Vit C	0020	04-7233	Aug. 2007	Aug. 2009	0.02	nd	0.02	Enugu
9	Gauze Vit C	GZ00152	04-3174	Feb. 2007	Feb. 2009	0.16	nd	0.06	Awka
10	Em-Vit C	807k	04-0262	Feb. 2007	Feb. 2009	0.13	nd	0.01	Lagos
11	Zvobes Cough Syrup	007	04-0665	Feb 2006	Feb. 2009	nd	nd	0.05	Lagos
12	Gauze multivitamine	00213	04-3234	Feb. 2007	Feb. 2009	0.13	nd	0.11	Awka
13	Gaviron	Gvm-0049	04-5131	Feb. 2007	Feb. 2010	0.79	0.08	1.64	Awka
14	Coflin	L1907	04-0540	Sep. 2007	Aug. 2009	nd	nd	0.01	Lagos
15	Avipol	ASV02	04-4744	Jun. 2007	May 2010	nd	nd	0.05	Ogun
16	Cypri Gold	260807	04-1627	Aug. 2007	Jul. 2009	0.02	nd	0.04	Lagos
17	Heamoglobin Tonic	001HV	04-0338	Jul. 2007	Jul. 2010	0.18	0.06	0.11	Lagos
18	Emzoron Tonic	4327k	04-1450	Jul. 2007	Jul. 2009	0.38	0.03	0.46	Lagos
19	Babyrex	TBB81	04-3937	Nov. 2007	Oct. 2010	nd	nd	0.02	Ilorin
20	Gauze Chloroquine	TN00151	04-3166	Jan. 2006	Feb. 2009	nd	nd	0.06	Awka
21	Septrin Syrup	6002	04-1888	Oct. 2006	Oct. 2009	nd	nd	nd	Ogun
22	Kp Multivitamine Syrup	HM2199M	NA	Feb. 2007	Jan. 2009	nd	nd	nd	Ogidi
23	Seven Keys Herbal Mixture	179*10/08	04-1986L	Oct. 2007	Oct. 2010	Nd	nd	0.47	Onitsha
24	Phenergan	LOTIW1200	04-0290	Jun. 2006	Aug. 2009	0.04	nd	0.02	Lagos
25	Magcid Suspension	MD0065	04-5136	Nov. 2007	Nov. 2009	4.13	0.01	1.90	Awka
26	Jawaron Syrup	L7019	04-2037	Jul. 2007	Jun. 2009	0.01	0.10	4.37	Lagos
27	Diastop Suspension	7011	04-1393	Jan. 2007	Jan. 2011	Nd	nd	0.37	Lagos
28	Priton Syrup	7k514008	04-0437	Oct. 2007	Sep. 2010	Nd	nd	0.07	Ogun
29	2.2.1 Forte Chloroquine Sulphate	LOT1X-120	04-1718	Jan. 2007	Dec. 2010	0.25	nd	nd	Lagos
30	Paracetamol Syrup	L124M	04-0289	Jan. 2008	Jan. 2011	Nd	nd	nd	Lagos
31	Tixylix	IW815	04-0320	Sep. 2006	Aug. 2009	0.22	nd	0.10	Lagos
32	Broncholyte	BI 63	04-2904	Dec. 2007	Dec. 2010	Nd	nd	nd	Ogun
33	Colipan	B 109	04-4044	Aug. 2007	Aug. 2010	Nd	nd	nd	Ogun
34	EM-B-Plex	3497k	04-0287	Jul. 2007	Jul. 2010	0.02	nd	nd	Lagos

Key: MFG, Manufacturing; Nd, Not Detected; NA, Not Available; NAFDAC, National Agency for Foods, Drugs Administration & Control

**Table 2.** Heavy metals (Cr, Ni and Mn) levels (mg/L) of imported pediatric syrup.

**S/No**	**Pharmaceutical Product**	**Batch No**	**NAFDAC NO**	**MFG Date**	**Expiry Date**	**Metal Level (mg/L)**			**Place of Mfg.**
						**Cr**	**Ni**	**Mn**	

1	Bellis Cough Syrup	9601	04-1814	Jun. 2006	Jun. 2009	nd	nd	0.02	Southport, England
2	MIM iron syrup	003705XP	04-5942	Jun. 2007	May 2010	nd	nd	0.05	Mubai, India
3	Pentax Paracetamol Syrup	L41006	04-0073	Oct. 2006	Sep. 2008	nd	nd	nd	Lagos, Nigeria^a^
4	Woodwards Gripe water	070048	04-1030	Aug. 2007	Aug. 2010	nd	nd	nd	Lagos, Nigeria^b^
5	Menthodex Cough Syrup	672H1	04-0971	Jun. 2007	Jun. 2010	nd	Nd	nd	United Kingdom
6	Chloramphenicol	71002	04-2745	Oct. 2007	Oct. 2010	nd	Nd	nd	Sango-Ota, Nigeria^c^
7	Erythromycin Suspension	007	04-5863	Nov. 2005	Nov. 2008	nd	Nd	nd	Bulchistan, Pakistan
8	Cephalexin Syrup	061624A	04-0883	Jan. 2006	Jan. 2009	0.01	Nd	nd	Cairo, A.R.E, Egypt
9	Vardorange Syrup	Vo-21	04-6063	Feb. 2006	Jan. 2009	nd	0.02	0.36	Mumbai, India
10	Ailon Multivitamin Drop	360345	04-3204	Nov. 2006	Oct. 2008	nd	0.03	0.03	London, England
11	Haemoglobin Syrup	37527	04-7608	Feb. 2006	Jan. 2010	nd	Nd	0.36	Tipperary, Ireland
12	Halfan	6005	04-2181	Mar. 2006	Mar. 2009	nd	Nd	Nd	Nanterre, France
13	Zentel Albendazole	370001	04-2467	Jan. 2007	Dec. 2009	nd	0.03	0.17	Bangalore, India
14	Maxiquine	L8107	04-5253	Jan. 2007	Dec. 2008	nd	0.09	0.11	Lagos, Nigeria^a^
15	Piccan	S724	04-2729	Jul. 2007	Jul. 2010	nd	Nd	0.11	Dublin, Ireland
16	Cadiphen	E 7015	04-1876	May 2007	Apr. 2011	nd	0.01	0.02	Dholka, India

^a^ licensed by Vitabiotic, England

^b^ licensed by Woodward, England

^c^ licensed by Omega Mayor, Jersey, UK

**Table 3.** Examples of intake calculation.

**True metal intake**	**Calculation**	**Total Intake of metal**

Ni	5 mL × 0.03 + 5mL × 4.30 + 5 mL × 0.79	25.6 mg/mL, Ni
Cr	5 mL × nd + 5 mL × 0.01 + 5 mL × 08	0.9 mg/mL, Cr
Mn	5 mL × 28.23 + 5 mL × 1.90 + 5 mL × 1.64	158.85mg/mL, Mn

(i.e., assumed syrup volume multiplied by heavy metal contaminant level for each of the three products: the volume of the syrup was assumed to be 5 mL each).
